# Genome sequencing and comparative genomic analysis of highly and weakly aggressive strains of *Sclerotium rolfsii*, the causal agent of peanut stem rot

**DOI:** 10.1186/s12864-021-07534-0

**Published:** 2021-04-16

**Authors:** Liying Yan, Zhihui Wang, Wanduo Song, Pengmin Fan, Yanping Kang, Yong Lei, Liyun Wan, Dongxin Huai, Yuning Chen, Xin Wang, Hari Sudini, Boshou Liao

**Affiliations:** 1 Key Laboratory of Biology and Genetic Improvement of Oil Crops, Ministry of Agriculture and Rural Affairs, P.R. China, Oil Crops Research Institute of the Chinese Academy of Agricultural Sciences, Wuhan, 430062 China; 2 College of Agronomy, Jiangxi Agricultural University, Nanchang, 330045 China; 3 International Crops Research Institute for the Semi-Arid Tropics (ICRISAT), Patancheru, Telangana 502324 India

**Keywords:** Comparative genomic analysis, PacBio sequel sequencing, Pathogenesis-related genes, *Sclerotium rolfsii*

## Abstract

**Background:**

Stem rot caused by *Sclerotium rolfsii* is a very important soil-borne disease of peanut. *S. rolfsii* is a necrotrophic plant pathogenic fungus with an extensive host range and worldwide distribution. It can infect peanut stems, roots, pegs and pods, leading to varied yield losses. *S. rolfsii* strains GP3 and ZY collected from peanut in different provinces of China exhibited a significant difference in aggressiveness on peanut plants by artificial inoculation test. In this study, *de-novo* genome sequencing of these two distinct strains was performed aiming to reveal the genomic basis of difference in aggressiveness.

**Results:**

*Scleotium rolfsii* strains GP3 and ZY, with weak and high aggressiveness on peanut plants, exhibited similar growth rate and oxalic acid production in laboratory. The genomes of *S. rolfsii* strains GP3 and ZY were sequenced by Pacbio long read technology and exhibited 70.51 Mb and 70.61 Mb, with contigs of 27 and 23, and encoded 17,097 and 16,743 gene models, respectively. Comparative genomic analysis revealed that the pathogenicity-related gene repertoires, which might be associated with aggressiveness, differed between GP3 and ZY. There were 58 and 45 unique pathogen-host interaction (PHI) genes in GP3 and ZY, respectively. The ZY strain had more carbohydrate-active enzymes (CAZymes) in its secretome than GP3, especially in the glycoside hydrolase family (GH), the carbohydrate esterase family (CBM), and the polysaccharide lyase family (PL). GP3 and ZY also had different effector candidates and putative secondary metabolite synthetic gene clusters. These results indicated that differences in PHI, secreted CAZymes, effectors and secondary metabolites may play important roles in aggressive difference between these two strains.

**Conclusions:**

The data provided a further understanding of the *S. rolfsii* genome. Genomic comparison provided clues to the difference in aggressiveness of *S. rolfsii* strains.

**Supplementary Information:**

The online version contains supplementary material available at 10.1186/s12864-021-07534-0.

## Background

*Sclerotium rolfsii* is a destructive soil-borne fungal pathogen. Its sexual stage, *Athelia rolfsii*, belongs to Basidiomycota and rarely occurs in nature; thus, its role in the life cycle of the fungus is unknown [1]. *S. rolfsii* infects more than 600 plant species, especially economically important agricultural and horticultural crops including peanut, soybean, wheat, cotton, tomato, potato, cucurbit, and onion [2, 3], therefore a pathogen of wide host range. Moreover, *S. rolfsii* produces sclerotia, which plays a key role in the disease cycle and can survive in soil for long periods [4]. *S. rolfsii* can infect stems, roots, pegs, and pods of peanut and causes branches wilting, and even whole plant wilting. Peanut stem rot caused by *S. roflsii* is also known as southern stem rot, southern blight, white mold, and *Sclerotium* rot [5]. This fungal disease has been reported in many peanut producing regions of the world. Loss caused by peanut stem rot was estimated at 41 million US dollars in Georgia in 2011 [6]. Up to 30% yield loss was recorded in India [7]. Peanut stem rot has been epidemic in China recently, caused up to 50% yield loss in hotspots, and is the most serious peanut soil-borne disease in China [8].

Control of peanut stem rot disease is difficult because of wide range of hosts, profuse mycelium, abundant persistent sclerotia, and genetic variability of *S. rolfsii* populations [4]. Currently, there are only a few resistant commercial peanut cultivars available for use [9–11]. Limited success was achieved in developing resistant varieties to peanut stem rot in China [12]. Normally, approaches to control peanut stem rot include the application of fungicides and agronomic measures such as rotation with non-host crops or coverage of infected crop debris with deep plowing [13]. But these methods are still not effective to control this disease.

In order to implement effective integrated practices to control peanut stem rot*,* knowledge about the genetic basis of differently aggressive strains of *S. rolfsii* is a key component, as it is essential for host resistance assessment in a given region [14]. Earlier investigators observed differences in aggressiveness among isolates of *S. rolfsii* in the USA and India [15–18]. They were classified as highly, moderately, and weakly aggressive strains [16]. Until now, differences in aggressiveness have not been reported among *S. rolfsii* strains in China. In previous research, aggressiveness of *S. rolfsii* strains were found to be highly correlated with endo-PG production and growth rate [16], but the genetic basis of aggressiveness is still unknown.

The genetic variability of *S. rolfsii* stains has not been documented. Correlations between pathogenic traits and genetic patterns have rarely been identified. To gain the relevant insights, we sequenced two *S. rolfsii* strains GP3 and ZY, GP3 isolated from Guangxi province and ZY isolated from Henan province, China, by combing the Single Molecule Real-Time (SMRT) sequencing and Illumina technology. The two strains were in different mycelial compatibility groups (MCG) [19], possessed similar cultural morphology and growth rate on PDA media, produced similar amount of oxalic acid *in vitro*, but demonstrated different levels of aggressiveness on peanut plants in inoculation tests. The ZY strain was highly aggressive, and the GP3 strain was weakly aggressive. In comparison with GP3 strain, ZY strain had a slightly larger genomes size. The genomes annotation of GP3 and ZY revealed that many pathogenesis- related genes differed between them, including pathogen host interaction (PHI) genes, CAZymes, secreted proteins and secondary metabolites. This study will be meaningful for further identifying determinants of pathogenicity as well as deeply understanding of *S. rolfsii* infection mechanisms.

## Results

### Aggressiveness, growth rate and OA production

**Fig. 1:**
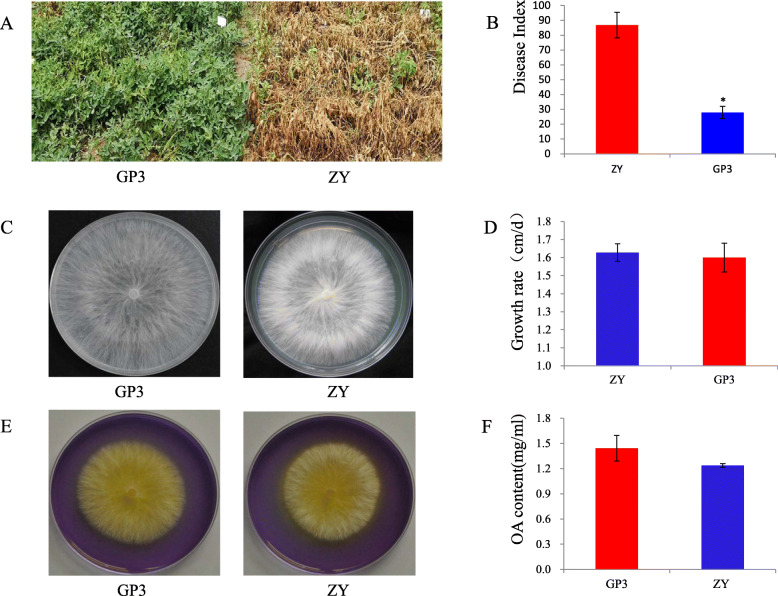
Pathogenicity, mycelial growth and oxalic acid production of *S. rolfsii* GP3 and ZY. **a** Symptom of peanut plants caused by GP3 and ZY; **b** Disease index of peanut plants infected by GP3 and ZY; **c** Mycelial growth of GP3 and ZY on potato dextrose agar (PDA) plates; **d** Growth rate of GP3 and ZY on PDA plates, **e** Mycelial growth of GP3 and ZY on PDA plates containing bromophenol blue; **f** Oxalic acid content of GP3 and ZY in PDB medium

The typical symptoms caused by *S. rolfsii* strains ZY and GP3 on the peanut stems included unrestricted lesions at the infection sites followed by tissue maceration, finally partial plant even whole plant wilting. Disease severity was scored at 14 days past inoculation (dpi) and disease index showed a significant difference between these two strains. The disease index of ZY was 82.34, which was classified as highly aggressive. The disease index of GP3 was 32.2, which was regarded as weakly aggressive (Fig. 1a, b). The growth rate of these two strains was similar on PDA plate and showed no significant difference (Fig. 1c, d). There was no significant difference in the amount of oxalic acid (OA) produced by these two strains either by haloes revealing on the PDA plate containing bromophenol blue, or by OA amount in the culture filtrate as analyzed by KMnO_4_ titration (Fig. 1e, f).

### Genome sequence and assembly

**Fig. 2:**
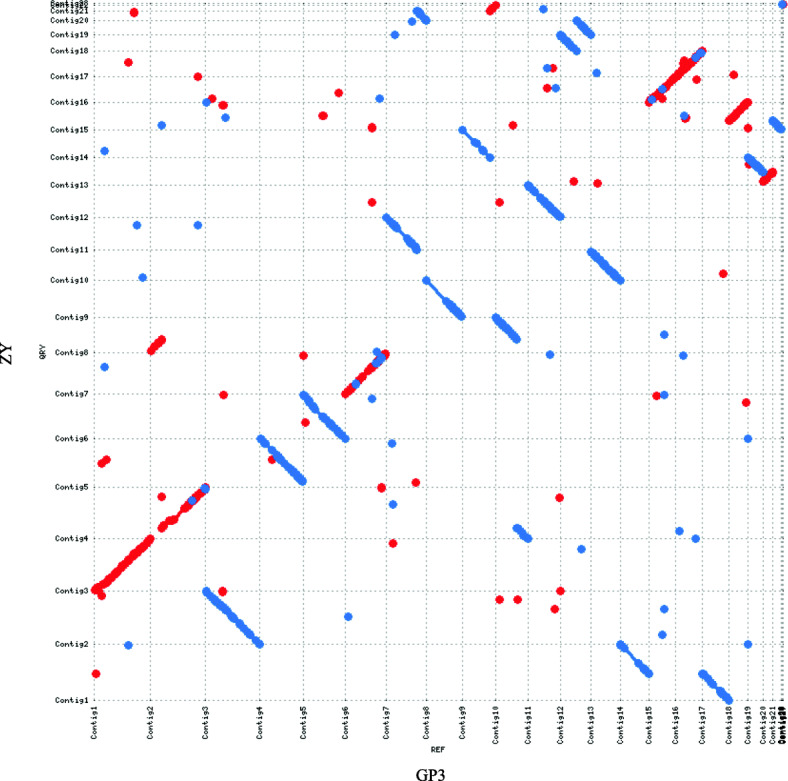
Genome synteny analysis between *S. rolfsii* strains GP3 and ZY. Dot-plots depicted nucleotide sequence matches detected via MUMer between all contigs of *S. rolfsii* GP3 and ZY. Contigs of ZY along the Y- axes, while contigs of GP3 along the X- axes. Sequence alignments exhibited a good macrosyntenic configuration

A total of 9.97 Gb subreads with 8.80 kb average length was generated for ZY and 6.34 Gb subreads with 10.68 kb average length for GP3 by SMRT sequencing. After polishing with Illumina data, the assembled genomes of GP3 and ZY were 70.51 Mb and 70.61 Mb, respectively, containing 27 contigs with an N50 of 3.67 Mb for GP3, and 23 contigs with an N50 of 3.71 Mb for ZY (Table 1). The two strains had genome assemblies of a similar size, both slightly smaller than that of *S. rolfsii* strain MR10 (73.18 Mb) [20]. The completeness of the genome assemblies was assessed using BUSCO [21]. About 97.5% (1301/1335) and 97.2% (1298/1305) of gene groups required for the correct assembly of Basidiomycota were present in GP3 and ZY, respectively (Fig. S1). The average GC contents of the resulting *S. rolfsii* genomes of GP3 (46.27%) and ZY (46.29%) were comparable to *S. rolfsii* MR10 (46.16%) (Table 1). Gene candidates in the *S. rolfsii* GP3 and ZY genomes were predicted by a combined approach, and 17,097 and 16,743 genes with an average gene length of 2013.91 bp and 2039.76 bp were identified (Table 1). Approximately 93.27% (15,947) of GP3 genes and 93.93% (15,727) of ZY genes could be annotated by non-redundant nucleotide and protein sequences in the Cluster of Orthologous Groups (KOG), Gene Ontology (GO), Kyoto Encyclopedia of Genes and Genomes (KEGG), Non-redundant Protein (NR), and Swiss-Prot databases (Fig. S2, 3, 4, 5). The number of genes predicted in *S. rolfsii* strains GP3 and ZY was similar with that in *S. rolfsii* strain MR10 (16,830 genes) (Table 1). In this study, we identified 356 tRNAs, 48 rRNAs and 32 snRNAs in the genome of GP3, and 415 tRNAs, 55 rRNAs and 32 snRNAs in the genome of ZY (Table S1). Comparison of gene orthologous with nine Agricomycetes fungi by OrthoMCL [22], GP3 and ZY shared a similarly low number of unique genes with 75 for GP3 and 37 for ZY distributed in 62 and 19 gene families (Table S2), respectively. Sequence comparison between contigs of whole-genome assemblies indicated a good macrosynteny between GP3 and ZY. Especially, contig 3, 7, 10, 15, 16, and 17 of GP3 corresponded well with contig 1, 6, 14, 18, 15, and 16 of ZY (Fig. 2).

**Table 1:**
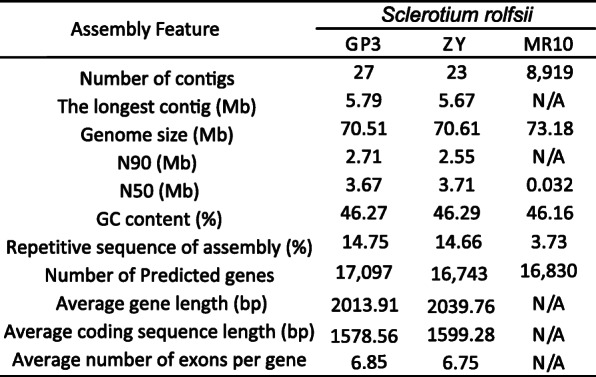
**Genome characteristic and assemblies feature of** ***S. rolfsii*** **strains.** *S. rolfsii* MR10 was the first sequenced *S. rolfsii* strain isolated from India. GP3 and ZY were isolated from China and sequenced in this study.

### Repetitive element analysis

**Fig. 3:**
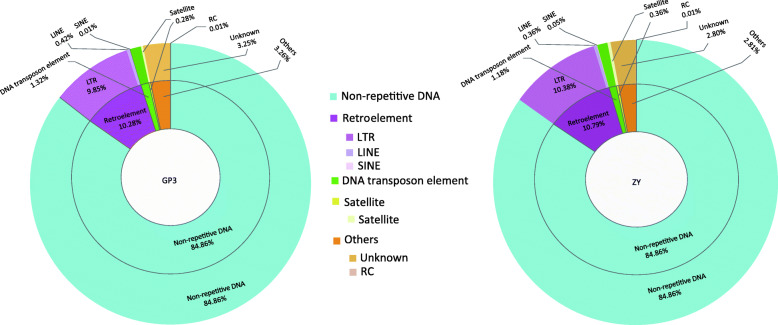
Distribution of repetitive sequences in *S. rolfsii* strains GP3 and ZY genomes. The left circle plot shows repetitive sequences distribution in *S. rolfsii* strain GP3, the right circle plot shows repetitive sequence distribution in *S. rolfsii* strain ZY. Repetitive sequence were classified as retroelement (LTR, long terminal repeat; LINE, long interspersed repeat element; SINE short interspersed repeat element), DNA transposon element, satellite, others, and non-repetitive element of genome

*De novo* and homology approaches were combined to identify repetitive sequences in the genomes of *S. rolfsii* GP3 and ZY. A total of 14.75% and 14.66% repetitive sequences were generated for GP3 and ZY, respectively (Table 1, Table S3). The abundance of repetitive sequences was similar between the two strains and much more than that of *S. rolfsii* strain MR10, which had a repetitive sequence content of 3.73% (Table 1). GP3 and ZY contained repetitive elements including DNA transposons, retroelements, and satellites. Retroelements were abundant in the studied genomes, accounting for 10.28% and 10.79% in GP3 and ZY. LTR was abundant in the retroelements, accounting for 9.85% and 10.38% in GP3 and ZY (Fig. 3, Table S4). Both abundance of LTR elements and retroelements in repetitive sequences were also found in *S. rolfsii* MR10 genome (Table S4).

### Orthology analysis and phylogenetic analysis

**Fig. 4:**
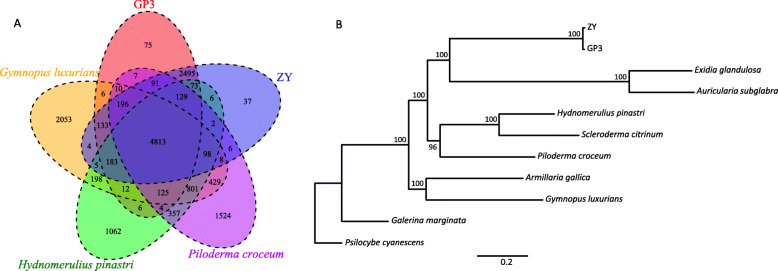
**Phylogenetic and comparative genomic study of** ***S. rolfsii*** **GP3 and ZY. a** Venn diagram showing an overlap of gene families among *S. rolfsii* GP3, ZY, *G. luxurians*, *P. croceum*, and *H. pinastri*; **b** Maximum likelihood phylogenetic tree of GP3, ZY and nine fungi species in Agaricomycetes based on single-copy orthologous genes, with *P. cyanescens* used as an outgroup species

The entire sets of predicted proteins of *S. rolfsii* GP3 and ZY were clustered with the OrthoMCL program [22] to identify gene families. Comparative analysis of the genomes of related species of Agaricomycetes, Basidiomycota showed that *S. rolfsii* strains had larger genomes but fewer total genes in comparison with most of the other species (Fig. S6). Of gene families, the unclustered genes number of GP3 and ZY were the least among fungi in Agaricomycetes. A Venn diagram of the OrthoMCL revealed that *S. rolfsii* strains shared 4813 genes with other four Agaricomycetes species (Fig. 4a).

To understand the genetic relationship of GP3 and ZY to the related Agaricomycetes species, we generated a phylogenetic tree of single-copy genes based the orthologous gene family analysis of the two *S. rolfsii* strains and other Agaricomycetes fungi, including *Armillaria gallica*, *Auricularia subglabra* [23], *Exidia glandulosa* [24], *Galerina marginata*, *Gymnopus luxurians* [25], *Hydnomerulius pinastri* [25], *Psilocybe cyanescens* [25], *Scleroderma citrinum* [25], and *Piloderma croceum* [25]*.* The phylogenetic tree indicated that *S. rolfsii* strains were more closely related to *E. glandulosa* and *A. subglabra,* which belonged to Auriculariales, than to *P. croceum*, which belonged to Atheliaceae, the same as *S. rolfsii* (Fig. 4b).

### Genes involved in pathogenicity

#### Homologs in PHI base

**Fig. 5:**
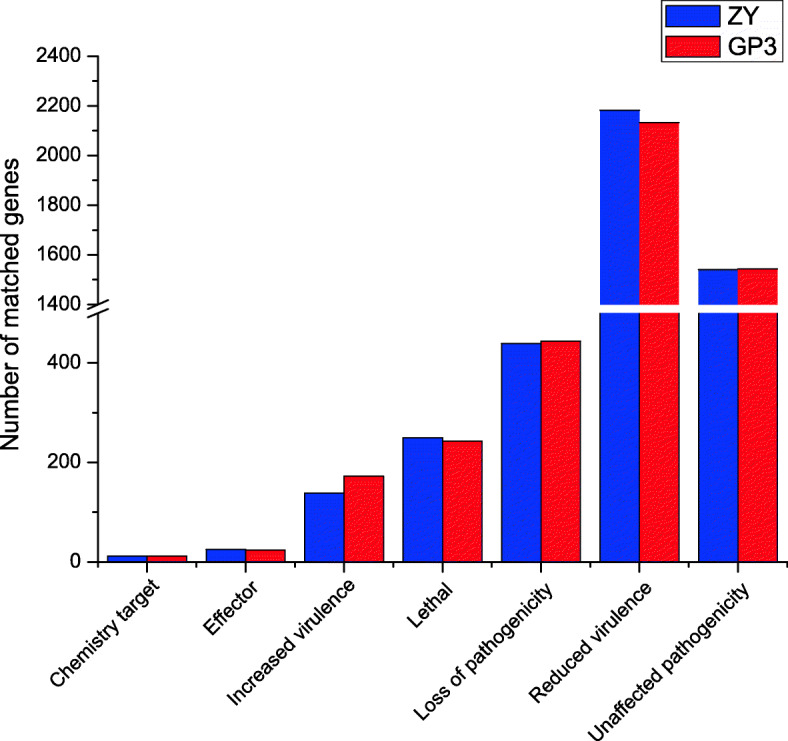
Pathogen-host interaction (PHI) genes of *S. rolfsii* GP3 and ZY. Distribution of *S. rolfsii* PHI genes in different phenotypes including chemistry target, effector, increased virulence, reduced virulence, lethal, loss of pathogenicity, and unaffected pathogenicity

In total, we identified 4600 and 4603 potential pathogen-host interaction (PHI) genes by searching the PHI base (Fig. 5). Among them, 24 genes were predicted as effector category and 172 genes were identified as “increased virulence” in GP3, while ZY had 25 effectors and 138 genes related to “increased virulence”. Compared with *S. rolfsii* GP3, a total of 45 genes were unique in ZY, two of which were predicted as effector and one was predicted as “increased virulence”. We also found 58 genes of GP3 were not present in the ZY genome, 12 and 18 of which were predicted as “loss of pathogenicity” and “reduced virulence”, respectively (Table S5).

#### CAZymes

**Fig. 6:**
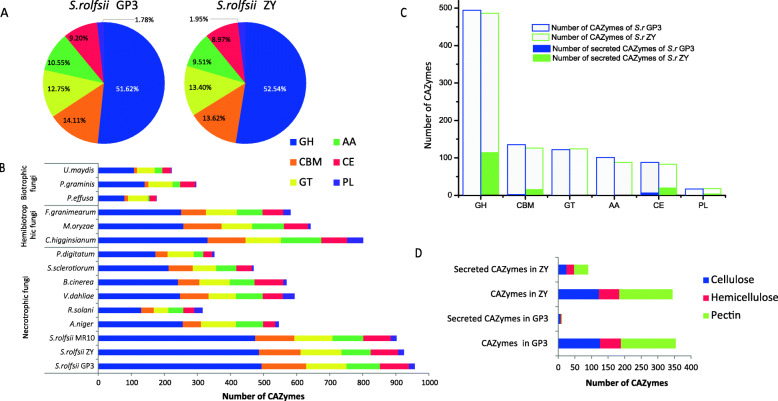
Distribution of CAZymes, secreted CAZymes, and CAZymes involved in plant cell wall degradation of *S. rolfsii* GP3 and ZY. **a** Distribution of CAZymes in *S. rolfsii* GP3 and ZY; **b** Comparison of CAZymes of *S. rolfsii* strains with other 12 plant pathogens; **c** Comparison of CAZymes and secreted CAZymes in GP3 and ZY; **d** Comparison of CAzymes and secreted CAZymes involved in plant cell wall degradation in GP3 and ZY. Abbreviations: GH, Glycoside hydrolase; CBM, Carbohydrate-binding module; GT, Glycosyltransferase; AA, Auxiliary activity; CE, Carbohydrate esterase; PL, Polysaccharide lyase

The genomes of *S. rolfsii* GP3 and ZY contained 957 and 925 genes encoding putative CAZymes, distributed in 118 and 119 CAZyme families. Glycoside hydrolases (GHs) were dominant in the GP3 and ZY genomes (51.62 and 52.54%), followed by carbohydrate-binding modules (CBMs) and glycosyltransferases (GTs) (Fig. 6a). The CAZyme content of GP3 was slightly larger than that of ZY, and CAZyme content of both GP3 and ZY was more than that of *S. rolfsii* MR10 (902) (Fig. 6b).

Comparison of CAZyme content of *S. rolfsii* strains with other plant pathogens including six necrotrophic fungi (*Aspergillus niger*, *Botrytis cinerea*, *Penicillium digitatum*, *Sclerotinia sclerotiorum*, *Rhizoctonia solani*, and *Verticillium dahliae*), three hemibiotrophic fungi (*Colletotrichum higginsianum*, *Fusarium graminearum*, and *Magnaporthe oryzae*), and three biotrophic fungi (*Puccinia graminis*, *Peronospora effusa*, and *Ustilago maydis*) showed that the CAZyme content of *S. rolfsii* genome was the highest among above analyzed pathogens (Fig. 6b). Necrotrophic fungi had more CAZymes than biotrophic and hemibiotrophic fungi. In comparison with those necrotrophic plant pathogens of a broad host range, such as *S. sclerotiorum*, *B. cinerea*, and *V. dahliae*, the CAZyme content of *S. rolfsii* was much more than these fungi. Compared to Basidiomycota plant pathogens, CAZyme content of *S. rolfsii* was three times as much as *R. solani* and *P. graminis*, and four times as much as *U. maydis* (Fig. 6b). Besides differences in CAZyme content, the number of CAZymes involved in cellulose, hemicellulose, and pectin degradation of *S. rolfsii* strains GP3 and ZY was noticeably larger than that of those analyzed pathogens (Tables S6–S8), especially in the pectin degrading capacity.

Glycoside hydrolases are known to catalyze the hydrolysis of glycosidic bonds in carbohydrate molecules. *S. rolfsii* was rich in one glycosyl hydrolase family, GH28, a class of polygalacturonases involved in pectin degradation. The amount of GH28 was the same in GP3 and ZY (62 vs 62) and was larger than that in the other analyzed pathogens (Table S8). The expansion of GH28 was not found in the biotrophic and hemibiotrophic pathogens, such as *U. maydis*, *P. graminis*, and *M. oryzae*. In comparison with other analyzed necrotrophic pathogenic fungi, *S. rolfsii* strains had three times more GH28. Besides GH28, some other glycoside hydrolases involved in pectin degradation in *S. rolfsii*, such as GH35, GH51, and GH78, also had higher number in comparison with those pathogens (Table S8).

#### Secretome and effector

The putative secreted proteins of *S. rolfsii* GP3 and ZY were identified based on a comprehensive pipeline (Fig. S7). The genomes of GP3 and ZY were predicted to encode 536 (3.14%) and 551(3.29%) secreted proteins, respectively. Among the secreted protein candidates, there were 151 and 30 secreted CAZyme genes for ZY and GP3, including 113 GH, 20 CE, 15 CBM, and 3 PL genes for ZY, while 22 GH, 6 CE, and 2 CBM genes for GP3 (Fig. 6c, Table S9). In comparison with secreted CAZymes involved in cellulose, hemicellulose and pectin degradation, ZY had more of these genes than GP3 (Fig. 6d).

A total of 50 and 46 putative effector candidates for GP3 and ZY, respectively, were predicted by Effector P.1. After manual inspection with the criteria of 50 ≤ molecular weight ≤ 300 kDa, 0–1 predicted trans-membrane domain, and ≥ 4 cysteine residues, a total of 30 and 27 effector candidates for GP3 and ZY were identified (Table 2). Most of the putative effector candidates were small (average length of 146 and 152 amino acids, ranging from 52 to 278, and 58 to 291 amino acids for GP3 and ZY). These candidates were rich in cysteines (the average cysteine composition was 8.5% for GP3 and 8.6% for ZY). The functions of most effector candidates (73.33% and 44.44% of GP3 and ZY) were unknown. Comparison of putative effectors with PHI and CAZymes candidate genes showed that the number of genes, for “functional effector”, “loss of pathogenicity”, “reduced virulence”, GH, and CBM, differed between these two strains. ZY had two effectors and five GH genes, while GP3 had one GH gene and no effector overlapping with PHI and CAZyme candidate genes (Table S10). The function of these predicted effectors needs to be further verified in future research.

**Table 2:**
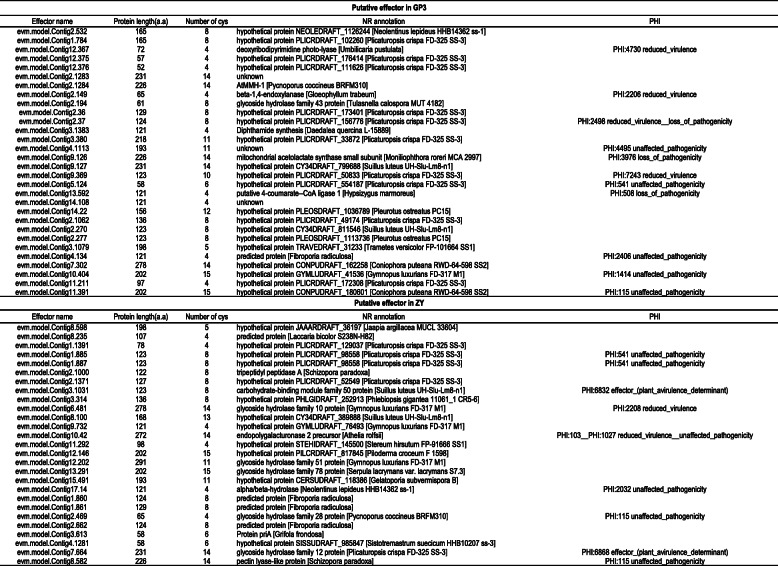
**Putative effectors of** ***S. rolfsii*** **GP3 and ZY.** Putative effectors of GP3 and ZY were functionally annotated in NCBI-NR and PHI base.

#### Secondary metabolites

**Fig. 7:**
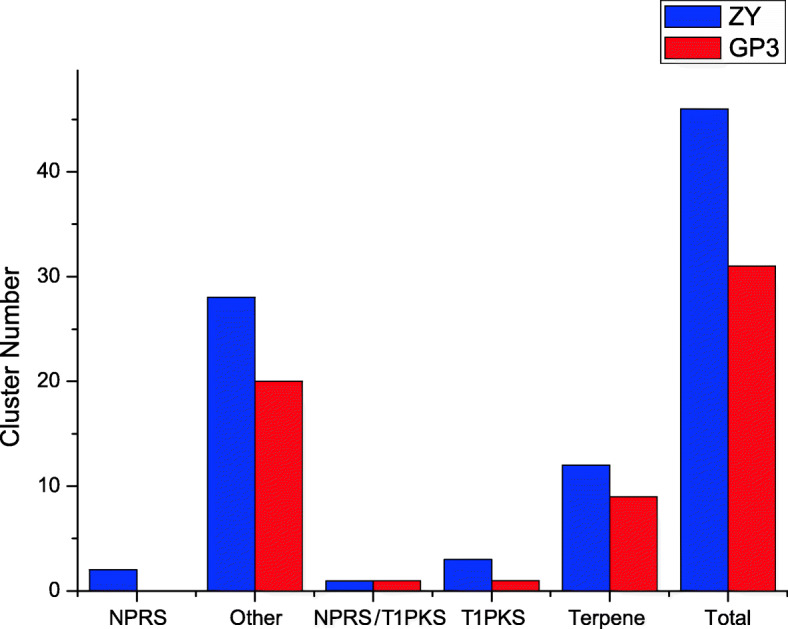
Putative secondary metabolite gene clusters in *S. rolfsii* ZY and GP3. Abbreviations: PKS, polyketide synthase; NRPS, nonribosomal peptides synthase; T1PKS, type 1 PKS; PKS-NRPS: hybrid NRPS-PKS enzymes

The antiSMASH 4.0 software was used to identify the secondary metabolite gene clusters in the genome of *S. rolfsii* ZY and GP3. A total of 46 and 31 gene clusters were predicted to be related to secondary metabolism in ZY and GP3, respectively (Fig. 7). In ZY, two clusters were identified as non-ribosomal peptide synthase (NPRS). Three, one, and 12 clusters were predicted as Type I polyketide synthase (T1 PKS), NPRS/ T1 PKS, and terpene, respectively. Besides, 28 clusters were predicted as others. Compared to ZY, GP3 contained no NPRS cluster, the same number of NPRS/ T1 PKS clusters, two fewer T1 PKS clusters, three fewer terpene clusters, and 8 fewer other clusters (Fig. 7).

## Discussion

*Sclerotium rolfsii* is a very important plant pathogen with a broad host range. To date, the genome of one strain MR10 with little information on its aggressiveness had been sequenced [20]. In the present study, we discovered two *S. rolfsii* strains that differed in aggressiveness on peanut plants. Meanwhile, the two strains did not show a significant difference in growth rate and oxalic acid production. Thus, we conducted genome sequencing of the two *S. rolfsii* strains and produced gapless high-quality genomes aiming to unravel the genomic basis underlying the difference in aggressiveness between the two strains.

During pathogenesis, *S. rolfsii* may produce cell wall degrading enzymes such as endo-polygalacturonase (endo-PG) [26, 27], cellulase [28], and polymethylagalacturonase [16] in conjunction with oxalic acid (OA) [16, 26]. Bateman and Beer [26] suggested that OA, pectinase, and cellulase acted synergistically in the destruction of host tissue by *S. rolfsii.* Secretion of OA and endo-PG concomitantly with rapid mycelial growth appeared to be the key requirements for establishing infection [16]. Earlier investigators observed differences in aggressiveness among isolates of *S. rolfsii* [16, 29, 30] and found that aggressiveness was highly correlated with endo-PG production and growth rate, provided a base level of OA [16]. OA was reported to have a positive correlation to the aggressiveness of *S. roflsii* isolates [30]. In contrast, OA was not correlated with the aggressiveness of *S. rolfsii* by Punja (1985) [16], who found that highly, moderately, and weakly aggressive strains produced similar amounts of oxalic acid. In the present study, we tested GP3 and ZY on the PDA plate containing bromophenol blue and measured the amount of OA produced in a liquid PDB medium. The results indicated that there was no significant difference in oxalic acid production between the weakly aggressive strain GP3 and the highly aggressive strain ZY, although oxalic acid is an essential aggressiveness factor for *S. sclerotiorum* [31]. *S. rolfsii* produced a basic level of oxalic acid to acidic environment that facilitates the optimal activity of certain sets of cell wall degrading enzymes and peptidases. However, OA was not the essential factor for difference in aggressiveness between *S.rolfsii* GP3 and ZY.

Plant cell walls are an important barrier that plants used to protect themselves from attacking by a range of organisms. Plant cell wall carbohydrates form a complex network of different polysaccharides that can be subdivided into three categories: cellulose, hemicellulose, and pectin. Plant pathogenic fungi employ diverse gene repertoires, including carbohydrate-active enzymes (CAZymes), to invade host plants and subvert host immune systems [32, 33]. CAZymes are known to play an important role in host-pathogen interactions and, along with effectors, are prime targets for studying aggressive factors in fungi [34, 35]. CAZyme families with potential roles in aggressiveness were examined in *S. rolfsii* strains GP3 and ZY. In our study, the CAZyme content in weakly aggressive strain GP3 was found to be slightly more than that in highly aggressive strain ZY. GP3 also had a noticeably higher number of enzymes in the AA, CBM, and CE families. GP3 and ZY had a similar number of enzymes involved in cellulose, hemicellulose, and pectin degradation, these results indicated that CAZyme content was not related to the difference in aggressiveness between *S. rolfsii* GP3 and ZY. We then undertook further analysis of the secreted CAZymes, which were involved in plant cell wall degradation that played an important role in phytopathogenic penetration of their hosts [36]. There was a significant difference in the levels of secreted CAZymes between GP3 and ZY. Highly aggressive strain ZY possessed three times more secreted CAZymes (105) than weakly aggressive strain GP3 (30). ZY also possessed more enzymes involved in pectin degradation, such as GH28. These results indicated that secreted CAZymes, especially polygalacutronases, may play an important role in different aggressiveness between *S. rolfsii* strains ZY and GP3. It was in accordance with the results of Punja (1985) [16], who reported that the aggressiveness of *S. rolfsii* was highly correlated with endo-PG production.

To establish infection, fungal plant pathogens secrete effector molecules that manipulate host physiology, including immune responses that are triggered when plant hosts sense invading pathogens [37–39]. Effectors have been discovered in multiple plant pathogenic fungi and exhibit numerous different functions depending on the fungal lifestyle. Necrotrophic fungi, which feed on dead tissue, often produce effectors that promote cell death, whereas biotrophic fungi, which require living tissue, produce effectors that prevent cell death [40–43]. In some soil-borne vascular necrotrophic pathogens with a broad range of host plants, effectors involved in aggressiveness have been identified. In *S. sclerotiorum*, about 70 effectors have been identified [44], a small, cysteine-rich secreted protein with a cyanoviron-N homology (CVNH) domain, attenuated aggressiveness when deleted [45]. A total of 127 putative effectors were identified in another broad host range necrotrophic pathogen *V. dahliae* [46]. Among them VdCP1 contributed to aggressiveness and triggered the host plant’s immune system [47]. Up to now, little experimental evidence for the existence of similar effector proteins was available for *S. rolfsii*. To identify putative effectors involved in aggressiveness, we searched the whole proteome of *S. rolfsii* and found that the effectors of GP3 and ZY were completely different. *S. rolfsii* existed as a multi-nuclear heterokaryon, in which individual cells may carry multiple nuclei [6]. The method for the stable transformation of *S. rolfsii* has not been available yet, and thus functional testing of pathogenic candidate genes in further studies will be challenging.

Despite the variety of pathogenicity-related mechanisms involved, accumulating evidence indicates that necrotrophic plant pathogens interact with their hosts in a manner much more subtle than originally considered and that signaling between them plays a significant role in the lifestyle of these pathogens [48]. The mechanism of differences in aggressiveness is complicated in plant pathogens. Besides secreted CAZymes and effectors, other factors may also be involved in aggressiveness on host plants. It was reported that the genomic islands might contribute to the expanded genetic diversity and aggressiveness of *V. dahliae* [49]. Aggressiveness-associated effectors were often found to be affected by both repeat activity and repeat-induced point mutations (RIP) in *Leptosphaeria maculans* and *S. sclerotiorum* [50, 51].

To understand the difference in aggressiveness of these two strains in genome, we presented here the gapless genome sequences of *S. rolfsii* GP3 and ZY. This work has provided important clues to factors involved in aggressive difference between these two *S. rolfsii* strains. The data will provide a useful foundation for further studies to understand the mechanism of *S. rolfsii* infection.

## Conclusions

We generated long-read PacBio reads and gapless genome assemblies of two *S. rolfsii* strains with different levels of aggressiveness on peanut plants and then implemented a comparative genomic analysis of these two strains. The genome of *S. rolfsii* ZY and GP3 encoded different pathogen related gene repertoires. The obtained GP3 and ZY genome assemblies and annotation represent the few available *Sclerotium* genome resources for studying the pathogenic mechanism of this fungus toward peanut.

## Methods

### Isolates and oxalic acid production

*Scleotium rolfsii* strain ZY was originally collected from Zhengyang county (114.34 °E, 32.38 °N) in Henan province, and strain GP3 was collected from Guiping city (101.49 °E, 36.34 °N) in Guangxi province of China. The two strains were in different mycelial compatibility group (MCG) and exhibited similar growth rate on potato dextrose agar (PDA medium: 200 g peeled and sliced potatoes boiled for 20mins, 20 g dextrose, adjusted to pH 7.0, 20 g agar, to make the final volume to 1000 ml with distilled water [19]. Oxalic acid was detected by two methods. PDA containing bromophenol blue was used to test oxalic acid in PDA plate. Mycelium discs of each strain were placed at the center of PDA medium containing 0.5 g/l of bromophenol blue and kept at 30 °C in the dark, four petri dishes for each strain. The diameter of yellow halo was measured after three days. KMnO_4_ titration was used to detect oxalic acid in liquid PDB. The strains grew in liquid PDB medium, three replicates of 150 ml flasks containing 30 ml of medium were included for each isolate, three discs were added to each flask, and the flasks were incubated without shaking at 30 °C in the dark. The culture of each strain was filtered through a Whatman No.1 filter paper after 5 days incubation. Oxalic acid (OA) content in 5 ml filtrate was determined using a KMnO_4_ titration method following the procedure of Kritzman’s [52].

### Pathogenicity test

The experiment was conducted as a completely randomized design in three replications. Each plot consisted of three rows with 15 susceptible peanut plants per row. The plants were inoculated 50–55 days after sowing. *S. rolfsii* inoculum was prepared just before inoculation. Oat grains were soaked in water for 4 h, sterilized at 121 °C for 30 mins twice after water removed. The fresh mycelium discs of *S. rolfsii* GP3 and ZY were transferred to the flasks containing sterilized oat grains, respectively. The oat grains culture maintained in the dark at 30 °C until surface of grains covered by *S. rolfsii* mycelium. The oat grains inoculum was mixed with equal amount of sterilized sand to ensure uniform delivery of inoculum. Each plant was inoculated with 2 g of *S. rolfsii* oat inoculum and sand mixture. The plots were watered to field capacity after inoculation. Disease symptoms were investigated 14 days after inoculation. A 1–5 scale for the severity of wilting according to Shokes’ method [53], where 1 = no symptom, 2 = lesions on stem only, 3 = up to 25% of the plant wilting, 4 = 26–50% of the plant wilting, and 5 = > 50% of the plant wilting. Disease index was calculated by using the following formula. DI = {[Σ (number of plants × corresponding diseases scale)] / (total number of plants × the maximum disease scale)} × 100. Different level of aggressiveness was determined according to Punja (1985) [16], high aggressiveness with DI more than 66.7, and weak aggressiveness showing DI less than 33.3.

### DNA and RNA purification

To prepare genomic DNA and RNA for sequencing, GP3 and ZY were cultured on PDA plates overlaid by cellophane films and maintained in the dark at 30 °C for 3–4 days. Mycelia were collected and grounded for DNA and RNA extraction. High-molecular-weight genomic DNA for single-molecule real-time (SMRT) was extracted using the SMRTbellTM Templated Prep Kit 1.0 (PACBIO). The genomic DNA for Illumina sequencing was extracted using a CTAB method as previously described [54]. Total RNA was extracted from mycelia using the TRIZOL Kit (Invitrogen, Carlsbad, CA, USA) following the manufacture’s protocol.

### Genome sequencing and assembly

For PacBio Sequel genome sequencing, high molecular weight genomic DNA (20 μg) was random sheared with Covaris- g-Tube with a goal of DNA fragments of approximately 20 kb and end-repaired according to the manufacturer’s instructions. A blunt-end ligation reaction followed by exonuclease treatment was performed to generate the SMRT Bell template, then the library was qualified and quantified using an Agilent Bioanalyzer 12 kb DNA Chip (Agilent Technologies, Santa Clara, CA, USA) and a Qubit fluorimeter (Invitrogen, Carlsbad, CA, USA). SMRT Bell cells were sequenced using the PacBio Sequel sequencing platform (Nextomics Biosciences, Co., Ltd., Wuhan, China). After adaptor removed and low quality reads filtered out, a total of 594,166 and 1,124,070 high quality reads covering 6,343,564,369 and 9,972,706,733 base pairs were generated for *S. rolfsii* strains GP3 and ZY, respectively.

For Illumina sequencing, about 100 μg of genomic DNA were sheared to ~ 180 bp using a Covaris LE instrument and adapted for Illumina sequencing on Illumina Hiseq Xten platform (San Diego, CA, USA) by NextOmics Biosciences. Illumina short reads were trimmed using Trimmomatic version 0.36 [55], read length for Illumina sequencing procedure for genomic DNA was 150 bp, a total of 6.42 Gb and 7.03 Gb clean data were yielded for GP3 and ZY, respectively.

The cDNA libraries were prepared by Illumina TreSeq RNA Sample Preparation Kit (Illumina, Inc., San Diego, CA, USA) and validated according to Illumina’s low-throughput protocol. The RNA-seq was conducted on an Illumina HiSeq 2500 Platform with 150 bp paired-end strategy.

A *de novo* genome assemblies of ZY and GP3 were generated with the PacBio Sequel reads using CANU pipeline (v1.5) [56] with default setting. The assemblies were adjusted using Arrow program, and polished using Illumina reads by Pilon [20]. Finally, the completeness of assemblies was evaluated using BUSCO [21].

### Repetitive elements analysis

Repetitive elements were identified by using different methods. Transposable elements were analyzed using four programs, two programs for de novo prediction, including Repeat Moldeler (https://www.repeatmasker.org/RepeatModeler) and LTR finder [57], and the database based programs Repeat Masker (https://www.repeatmasker.org/) and Repeat-ProteinMasker (submodule in Repeatmasker) with default parameters to search Repbase [58]. Tandem Repeats Finder (TRF) was used to identify tandem repeat sequences [59]. MicroSAtellites (MISA) (https://www.plob.org/tag/misa) was used to identify simple sequence repeats (SSR) with default setting.

### Genome annotation

Gene predication was performed by using a combination of ab initio-based and homology-based methods. To aid gene annotation, we generated transcript assemblies based on RNA of GP3 and ZY, respectively. For ab initio-based prediction, Augustus v2.4 [60] and Genscan (version 1.0) [61] were used to de novo predict protein coding genes with the default setting. Exonerate [62] was used to predict the gene structure with RNA-seq data. For homology-based predication, GeneWise [63] was used to predict protein coding genes by homology analysis with known protein sequences from six related species of Basidiomycota, including *Galerina marginata*, *Gymnopus luxurians*, *Hydnomerulius pinastri*, *Jaapia argillacea*, *Piloderma croceum*, and *Plicaturopsis crispa*. EvidenceModler (EVM) was used to compute the weighed consensus gene structure annotation [64]. The final gene sets were obtained after removed TE transposable elements by Tranposon PSI [65].

The predicted gene sets of *S. rolfsii* GP3 and ZY were functionally annotated based on similarity comparison with homologous in public databases. BLASTP was used to align the protein sequences by automated searches in NCBI-NR, Swiss-Prot (https://www.expasy.org/sprot/), KEGG, GO and KOG database with E-values≤1e-5. Gene function domain annotation was conducted by InterProScan program [66]. The pathway analyses were conducted by KAAS-KEGG Automatic Annotation Serve [67]. The candidate non-coding RNA (ncRNA) was annotated by two approaches, BLAST was used to align the *S. rolfsii* genome against the Rfam database [68], and tRNA scan-SE [69] and RNAmmer [70] were used to predict tRNAs and rRNAs, respectively.

### Analysis of orthologous gene families in Agaricomycetes

Orthology comparison was conducted by OrthoMCL [22] (http://va.orthomcl.org) with e-value less than le-5 among protein sets of *S. rolfsii* GP3, ZY and nine related species of Agaricomycetes, including *Armillaria gallica* (GenBank: GCA_002307695.1), *Auricularia subglabra* (GenBank: GCA_000265015.1), *Exidia glandulosa* (GenBank: GCA_001632375.1), *Galerina marginata* (GenBank: GCA_000697645.1), *Gymnopus luxurians* (GenBank: GCA_000827265.1), *Hydnomerulius pinastri* (GenBank: GCA_000827185.1), *Psilocybe cyanescens* (GenBank: GCA_002938375.1), *Scleroderma citrinum* (GenBank: GCA_000827425.1), and *Piloderma croceum* (GenBank: GCA_000827315.1).

### Phylogenetic analysis and synteny analysis

The phylogenetic tree of *S. rolfsii* GP3, ZY and the above related nine species of Agaricomycetes was constructed by single copy gene based on the orthologous gene family analysis. Mafft [71] software was conducted to align the protein sequence of the single copy gene, converted to coding sequence alignment. Gblocks [72] was used to extract the well-aligned regions of each coding sequence alignments. RAxML 8.2.12 [73] was carried out to generate the maximum-likelihood tree with 100 bootstrap replicates with *P. cyanescens* as an outgroup. The whole genome aligner Murmer 3.06 [74] was used for comparative analysis of the assemblies of GP3 and ZY. Dot plots between contigs of GP3 and ZY were created by MuMerplot programs from the MuMmer package.

### Identification of the pathogenicity related genes

The *S. rolfsii* GP3 and ZY protein sets were used to conduct a BLASTP search against PHI base (a database of Pathogen Host Interactions) with e-value less than 1e-5 to identify pathogenicity genes. Putative carbohydrate active enzymes (CAZymes) of *S. roflsii* GP3 and ZY were annotation using dbCAN (dbCAN HMMs 5.0) [75] servers, with an e-value of less than 1e-5 and more than 70% coverage. CAZymes were classified by following modules: Glycoside Hydrolases (GHs), Polysaccharide Lyases (PLs), Carbohydrate Esterases (CEs), Glycosyl Transferase (GTs), Carbohydrate-Binding Modules (CBMs), and Auxillary Activities (AAs) as described in CAZyme database classification (http://www.cazy.org) [76].

### Secretome and effector predication

The predicted secretome of *S. rolfsii* strains GP3 and ZY was conducted based on the following pipeline. SignalP version 4.0 [77] was used to analyze signal peptide and cleavage sites of *S. rolfsii* GP3 and ZY proteins. Candidate proteins with signal peptide were identified by Protcomp 9.0 (http://www.softberry.com/berry.phtml) using the LocDB and PotLoc DB databases and proteins predicted as extracellular or unknown were kept for next analysis. The candidate proteins were conducted by TMHMM version 2.0 [78] to identify protein with transmembrane domains, and all proteins with 0 TM or 1 TM, if located in the predicted N- terminal signal peptide, were kept. The candidate proteins that harbored a putative glycophosphatidylinositol membrane-anchoring domain were identified by GPIsom (https://gpi.unibe.ch/) [79]. The remaining proteins without GPI-anchor were predicted with Target P [80], proteins with a Target P Loc = S or – were kept in the final secretome databases. The candidate secretory proteins were blasted in NR database and PHI database to annotate the protein function and searched against CAZyme database for function of CAZymes. The candidate effectors were identified by passing the secretome through the program Effector P 1.0 [81]. Putative effectors were screened for those candidates with molecular weight ranged from 50 to 300 amino acids, and at least 4 cysteine amino acids in their sequences [82–84].

### Secondary metabolites synthetic gene cluster predication

Secondary metabolites synthetic gene clusters were predicted by the web-based software antiSMASH (antibiotics and Secondary Metabolite Analysis 4.0) [85].

## Supplementary Information

**Additional file 1: Figure S1.** Statistics of BUSCO assessment of *S. rolfsii* GP3 and ZY genome assemblies. **a** Total searched BUSCOs of *S. rolfsii* GP3 and ZY; **b** Distribution of different BUSCOs in GP3 and ZY. S, Complete and single-copy BUSCOs; D, Complete and duplicated BUSCOs; F, Fragmented BUSCOs; M, Missing BUSCOs

**Additional file 2: Figure S2.** Genome annotation statistics of *S. rolfsii* GP3 and ZY by blasting against five databases including GO, KOG, KEGG, NR and Swiss_pro

**Additional file 3: Figure S3.** KOG distribution of predicted proteins of *S. roflsii* GP3 and ZY

**Additional file 4: Figure S4.** Go annotation enrichment analysis of genes of *S. rolfsii* GP3 and ZY. **a** Number of genes in biological process, cellular component, and molecular function of *S. rolfsii* GP3 and ZY; **b** Percent of genes involved in molecular function, biological process, and cellular component of *S. rolfsii* GP3; **c** Percentage of genes involved in molecular function, biological process, and cellular component of *S. rolfsii* ZY

**Additional file 5: Figure S5**. KEGG classification of genes of *S. rolfsii* GP3 and ZY. **a** Distribution of genes among processes, metabolism, and organismal systems of *S. rolfsii* GP3; **b** Distribution of genes among processes, metabolism, and organismal systems of *S. rolfsii* ZY

**Additional file 6: Figure S6.** Analysis of orthologs of two *S. rolfsii* strains and other species in Agaricomycetes

**Additional file 7: Figure S7.** The pipeline for putative secretomes and effectors analysis of *S. roflsii* GP3 and ZY

**Additional file 8: Table S1.** List of ncRNAs in genomes of *S. rolfsii* GP3 and ZY

**Additional file 9: Table S2.** Comparison of gene families of GP3, ZY, and other species in Agaricomycetes

**Additional file 10: Table S3.** Identification of repetitive elements of *S. rolfsii* GP3 and ZY by five programs

**Additional file 11: Table S4.** List of repetitive elements of each category in the genomes of *S. rolfsii* GP3, ZY, and MR10

**Additional file 12: Table S5.** Unique Pathogen Host Interaction (PHI) genes in the genome of *S. rolfsii* GP3 and ZY

**Additional file 13: Table S6.** Comparison of CAZymes involved in cellulose degradation in the genomes of plant pathogens. The values reflect the total numbers in each family of CAZymes.

**Additional file 14: Table S7.** Comparison of CAZymes involved in hemicellulose degradation in the gnomes of plant pathogens. The values reflect the total numbers in each family of CAZymes.

**Additional file 15: Table S8.** Comparison of CAZymes involved in pectin degradation in the genomes of plant pathogens. The values reflect the total numbers in each family of CAZymes.

**Additional file 16: Table S9.** List of secreted CAZymes in the genome of *S. rolfsii* GP3 and ZY

**Additional file 17: Table S10.** Overlapping of putative effectors with PHI and CAZymes genes

## Data Availability

The genomes of *S. rolfsii* strains GP3 and ZY were deposited in GenBank under BioProject numbers: PRJNA635225, PRJNA635226, and BioSample numbers: SAMN15029893, SAMN15029894, respectively.

## Abbreviations

AA: Auxiliary activity
*A. subglabra*: *Auricularia subglabra*
*B. cinerea*: *Botrytis cinerea*
CAZyme: Carbohydrate-active Enzyme
CBM: Carbohydrate-binding module
CE: Carbohydrate esterase
endo-PG: Endo-polygalacturonase
*E. glandulosa*: *Exidia glandulosa*
GH: Glycoside hydrolase
*G. luxurians*: *Gymnopus luxurians*
GPI: Glycosylphosphatidylinositol anchor
GT: Glycosyltransferase
*P. croceum*: *Piloderma croceum*
*P. cyanescens*: *Psilocybe cyanescens*
*P. tinctorius*: *Pisolithus tinctorius*
PL: Polysaccharide lyase
PHI: Pathogen- host interaction
*P. graminis*: *Puccinia graminis*
*R. solani*: *Rhizoctonia solani*
*S. rolfsii*: *Sclerotium rolfsii*
*S. sclerotiorum*: *Sclerotinia sclerotiorum*
*U. maydis*: *Ustilago maydis*
*V. dahliae*: *Verticillium dahliae*
