# Supplementary figures and images for: Genome sequencing and comparative genomic analysis of highly and weakly aggressive strains of Sclerotium rolfsii, the causal agent of peanut stem rot
Source: BMC Genomics. 2021 Apr 16;22:276. doi: 10.1186/s12864-021-07534-0 (PMC8052761; doi:10.1186/s12864-021-07534-0)

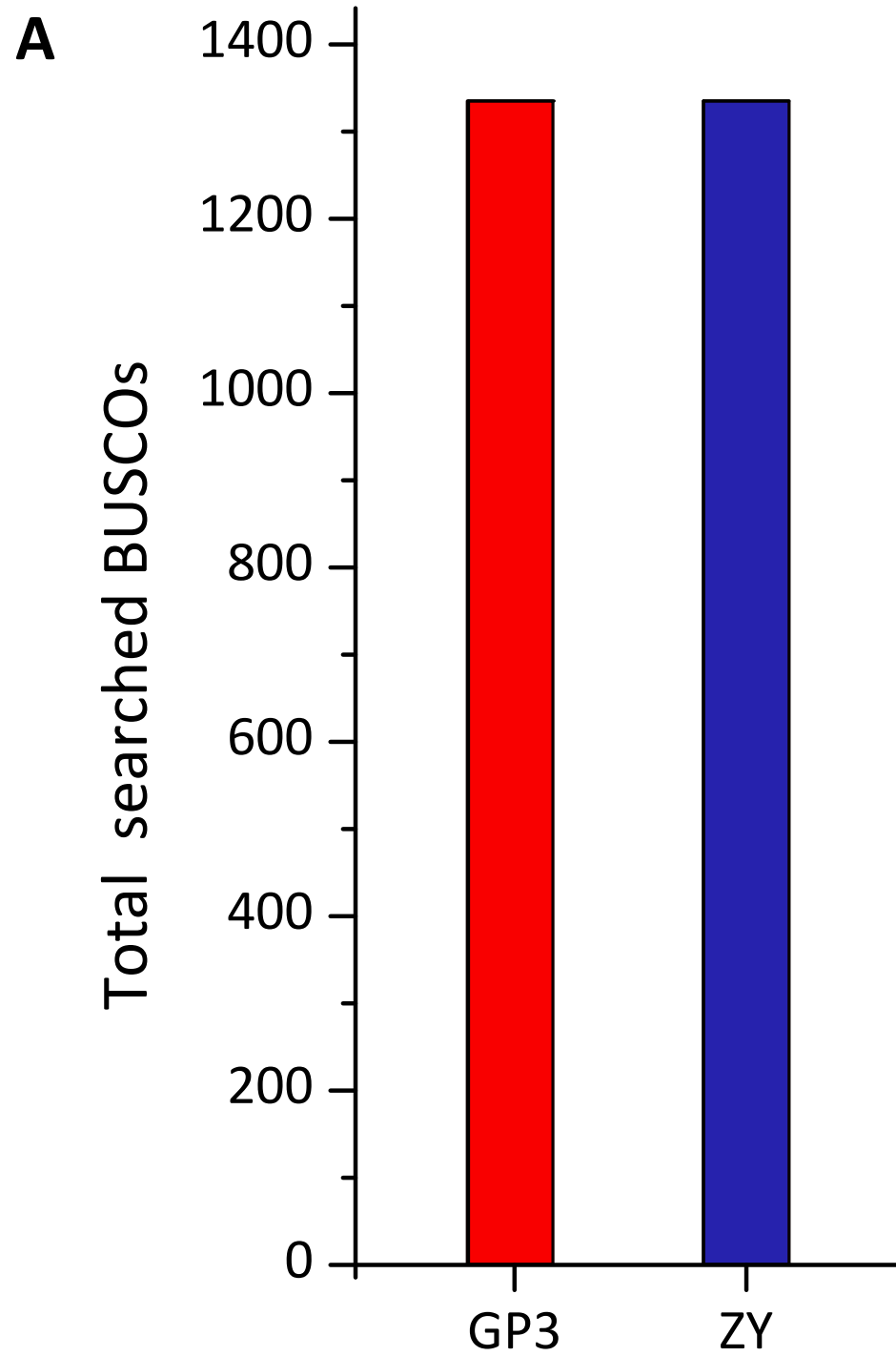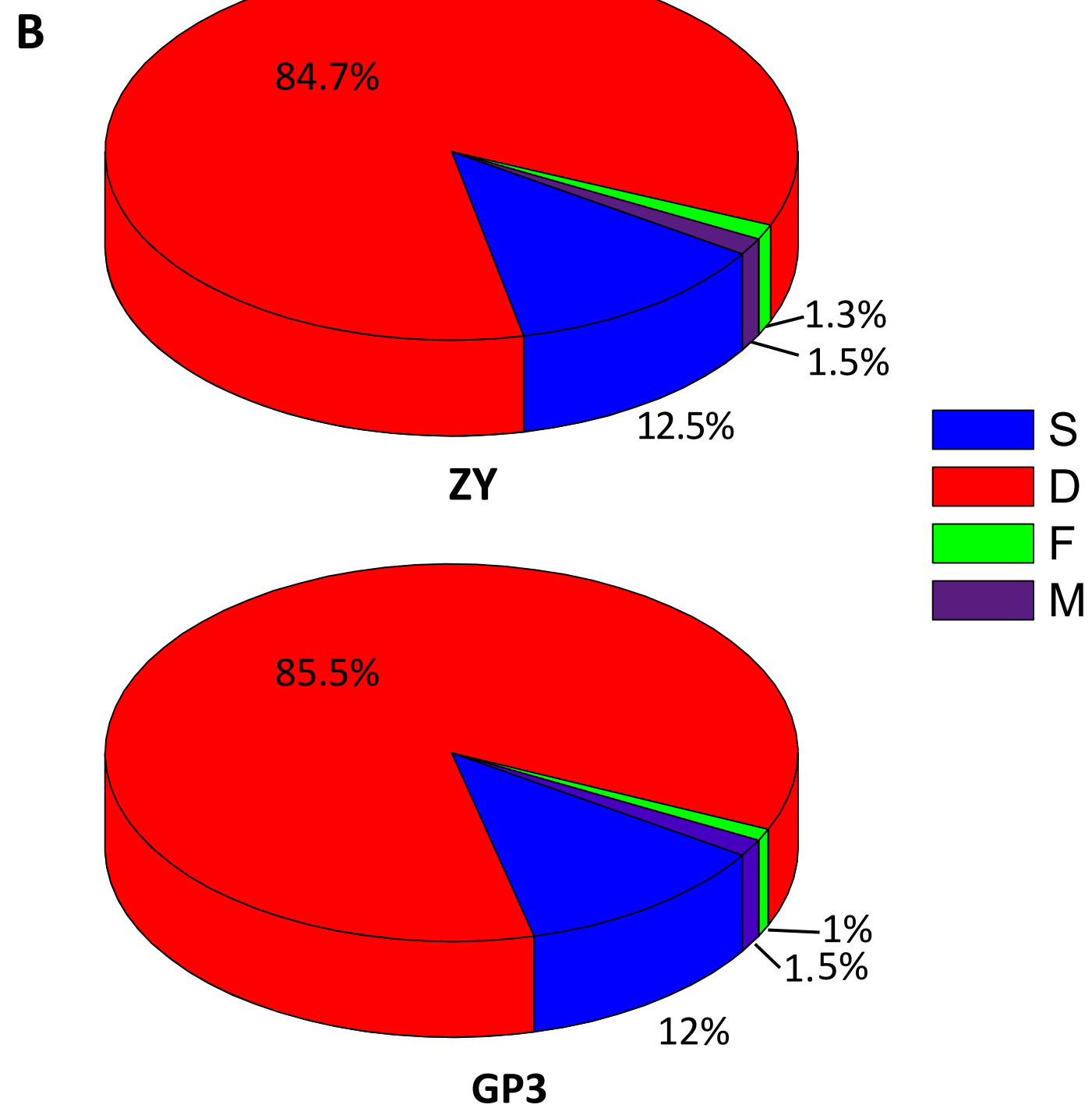

Supplement: Supplementary file 1 — Additional file 1: Figure S1. Statistics of BUSCO assessment of S. rolfsii GP3 and ZY genome assemblies. a Total searched BUSCOs of S. rolfsii GP3 and ZY; b Distribution of different BUSCOs in GP3 and ZY. S, Complete and single-copy BUSCOs; D, Complete and duplicated BUSCOs; F, Fragmented BUSCOs; M, Missing BUSCOs [file 12864_2021_7534_MOESM1_ESM.pdf]

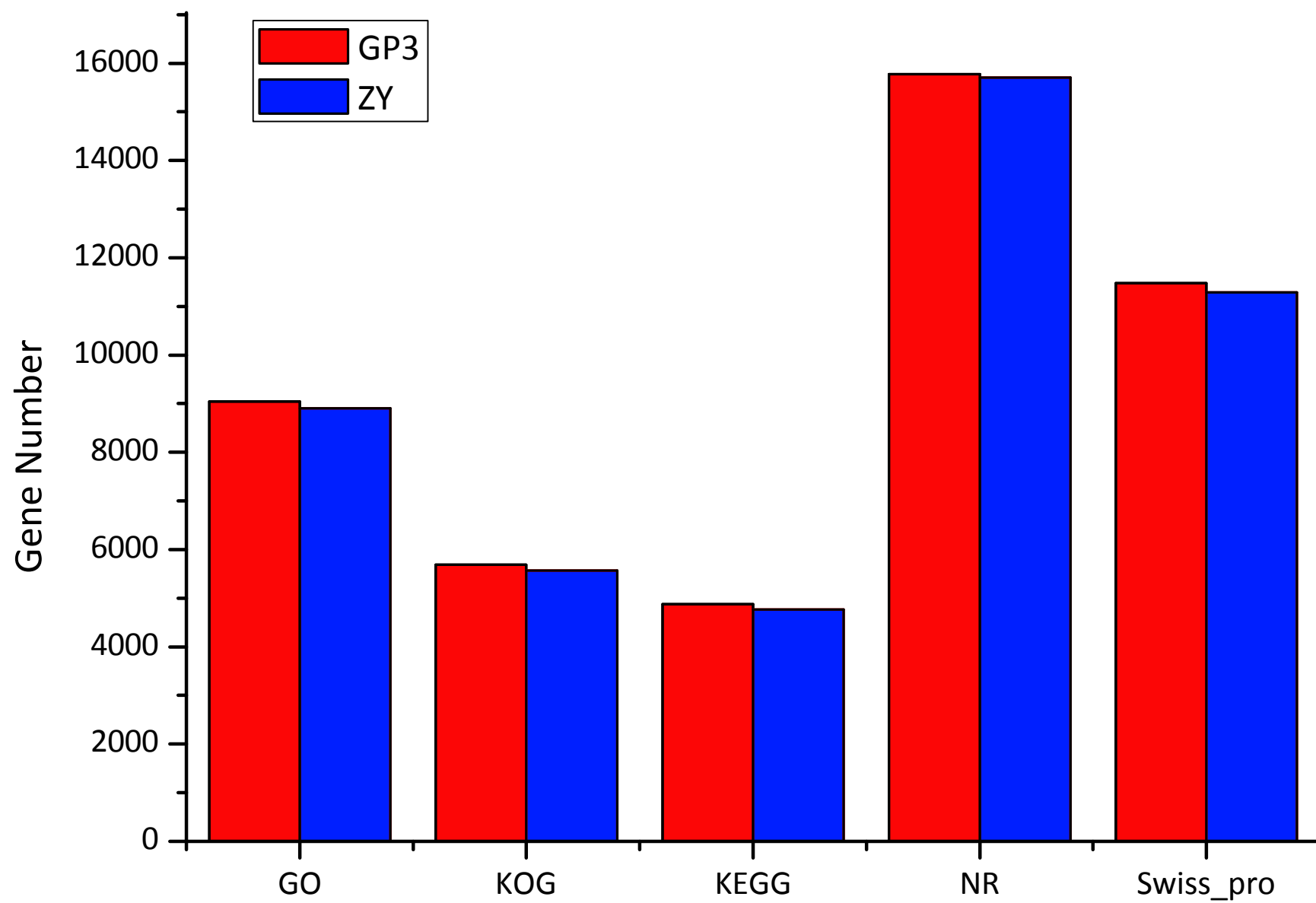

Supplement: Supplementary file 2 — Additional file 2: Figure S2. Genome annotation statistics of S. rolfsii GP3 and ZY by blasting against five databases including GO, KOG, KEGG, NR and Swiss_pro [file 12864_2021_7534_MOESM2_ESM.pdf]

A

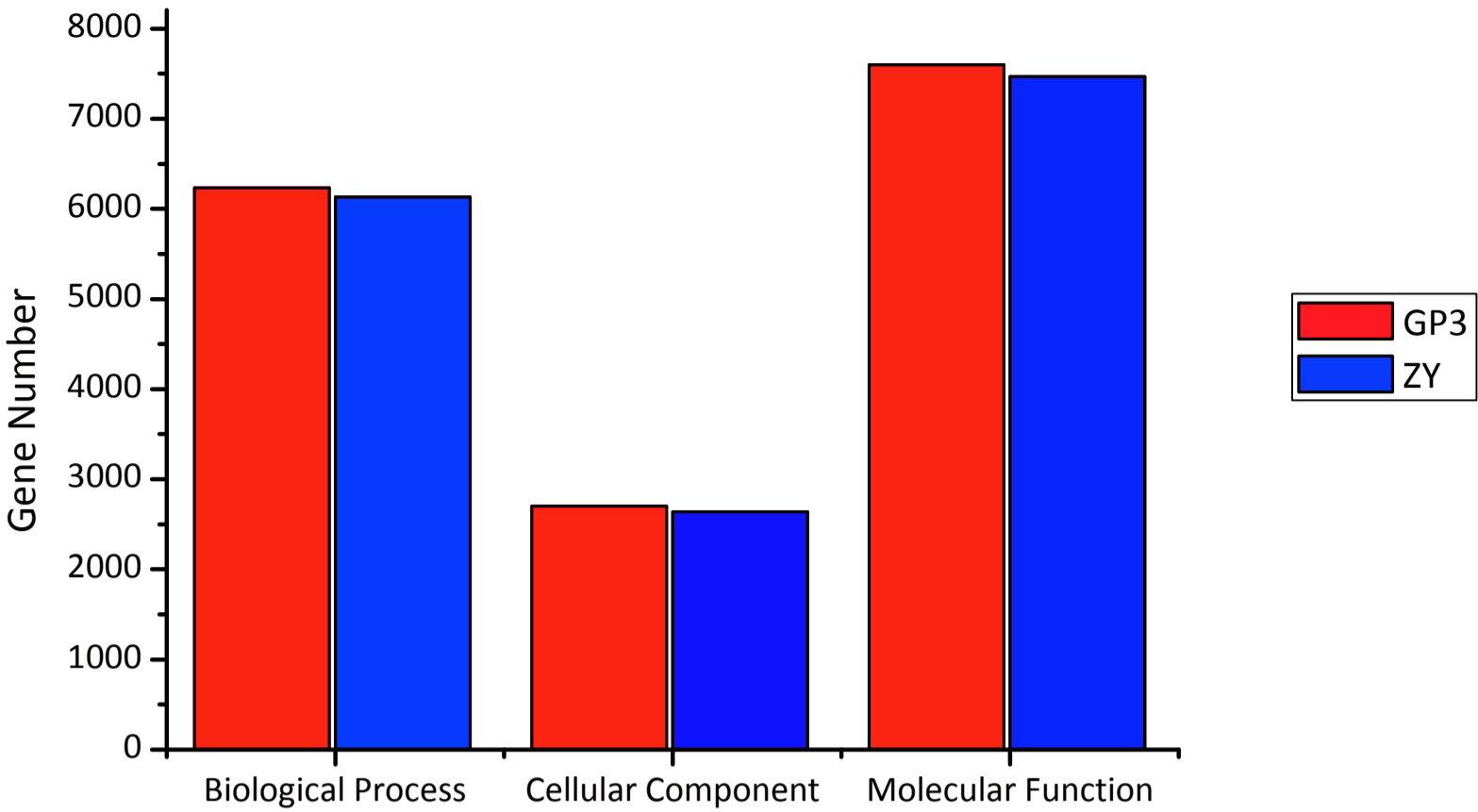

B

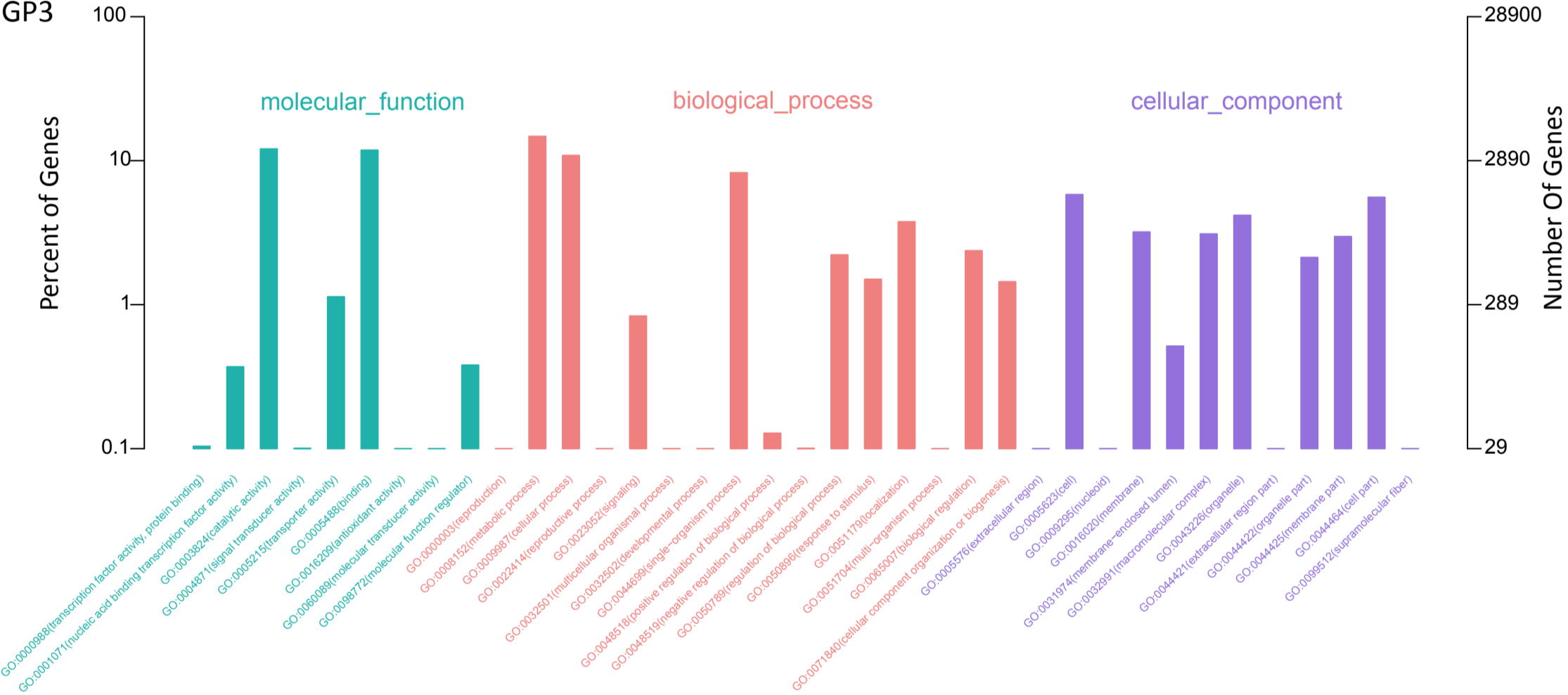

C

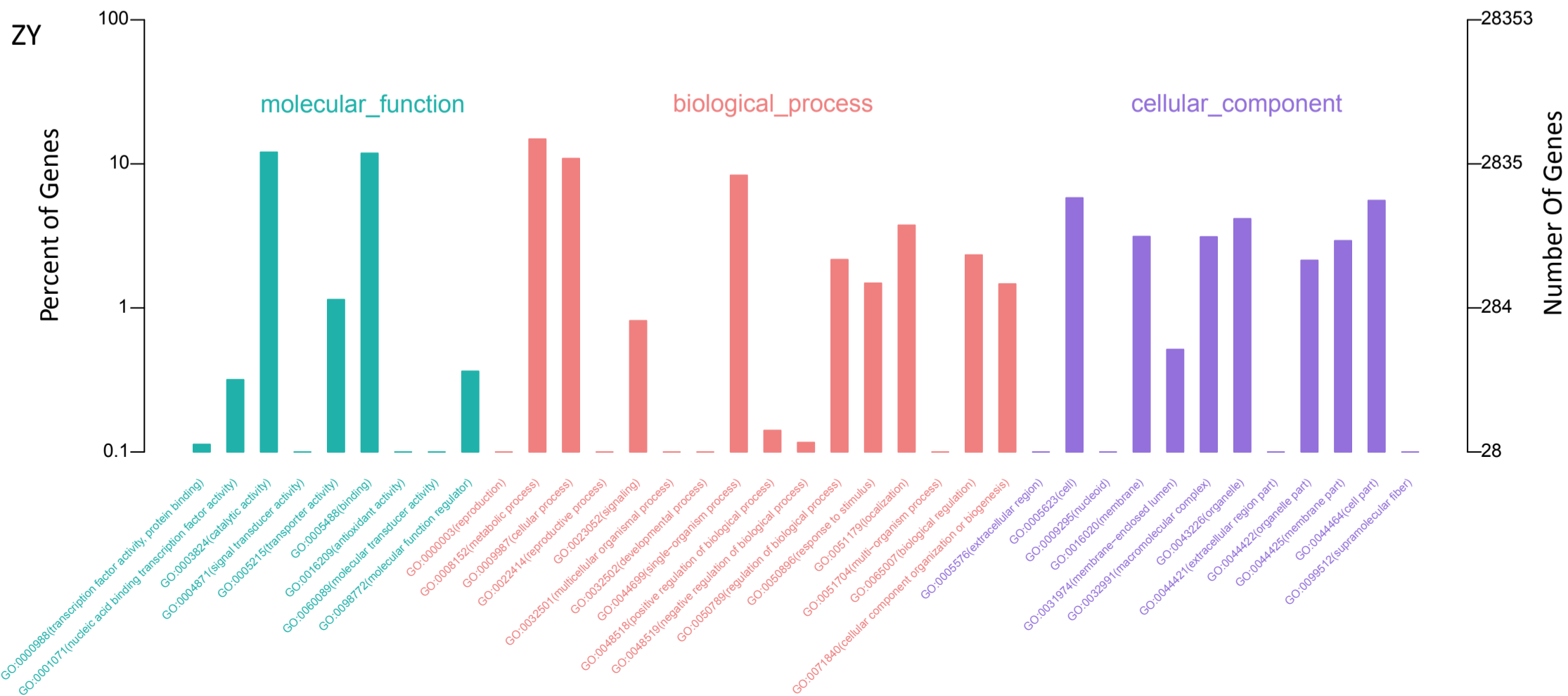

Supplement: Supplementary file 4 — Additional file 4: Figure S4. Go annotation enrichment analysis of genes of S. rolfsii GP3 and ZY. a Number of genes in biological process, cellular component, and molecular function of S. rolfsii GP3 and ZY; b Percent of genes involved in molecular function, biological process, and cellular component of S. rolfsii GP3; c Percentage of genes involved in molecular function, biological process, and cellular component of S. rolfsii ZY [file 12864_2021_7534_MOESM4_ESM.pdf]

A

GP3 KEGG Classification

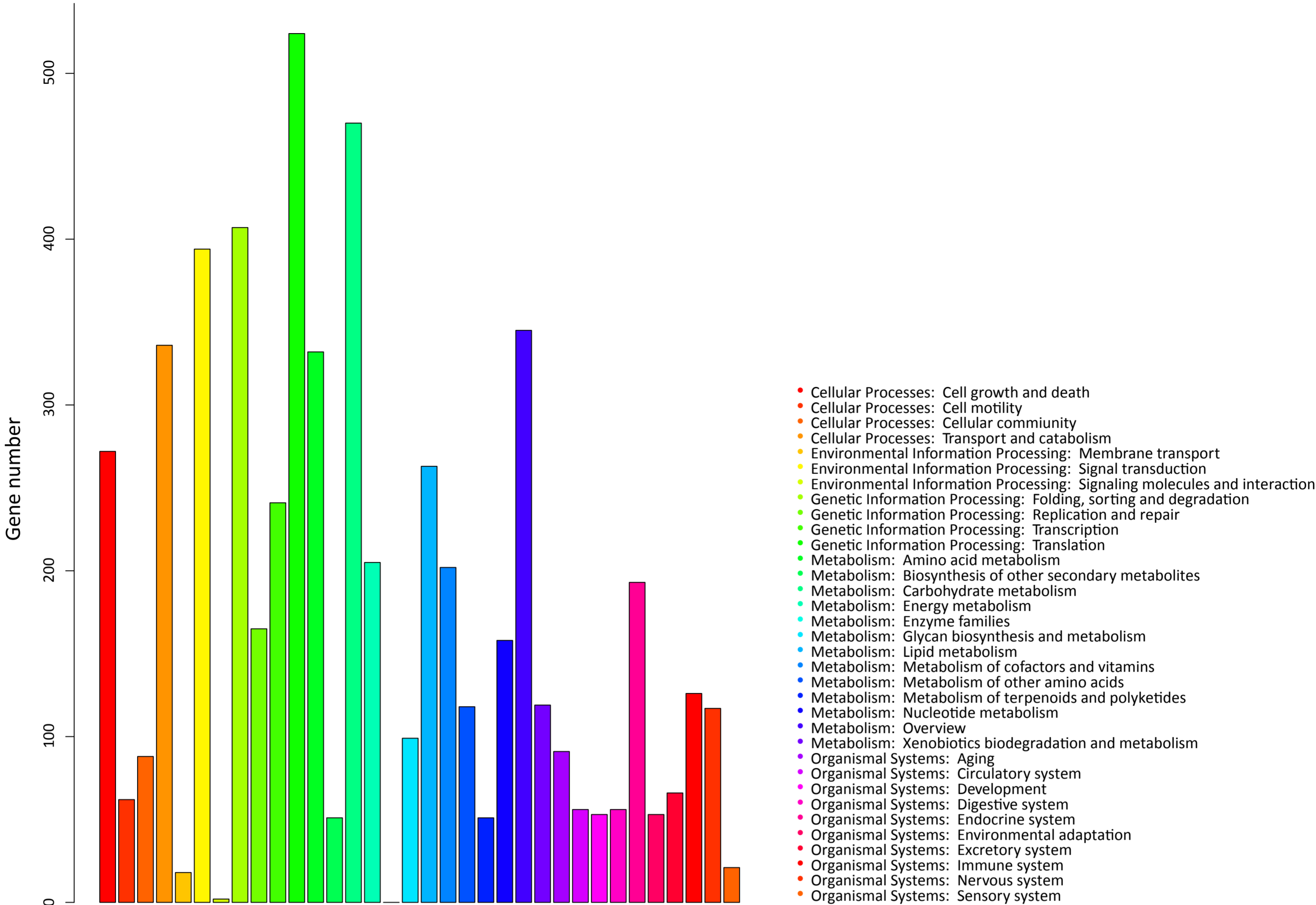

B

ZY KEGG Classification

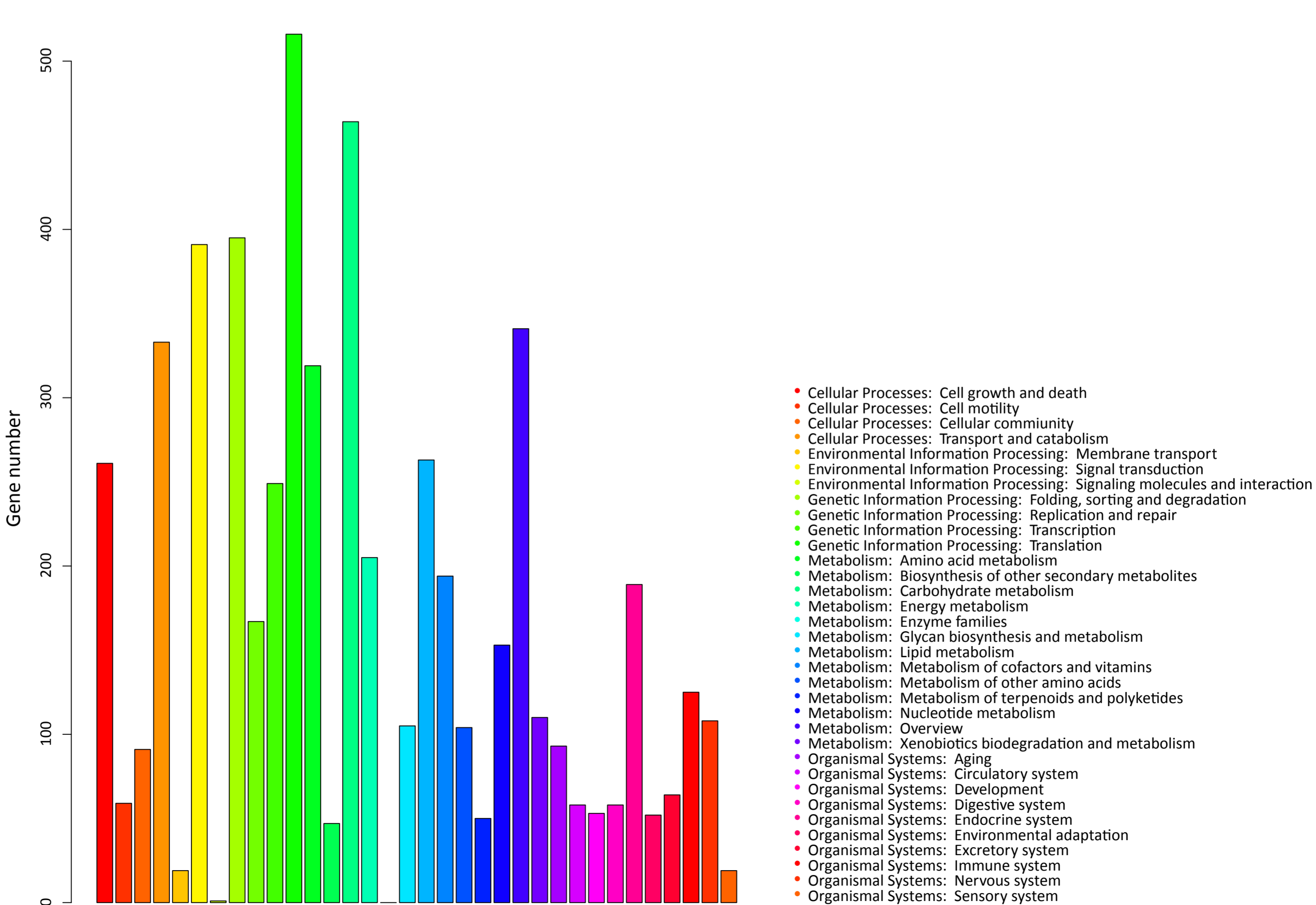

Supplement: Supplementary file 5 — Additional file 5: Figure S5. KEGG classification of genes of S. rolfsii GP3 and ZY. a Distribution of genes among processes, metabolism, and organismal systems of S. rolfsii GP3; b Distribution of genes among processes, metabolism, and organismal systems of S. rolfsii ZY [file 12864_2021_7534_MOESM5_ESM.pdf]

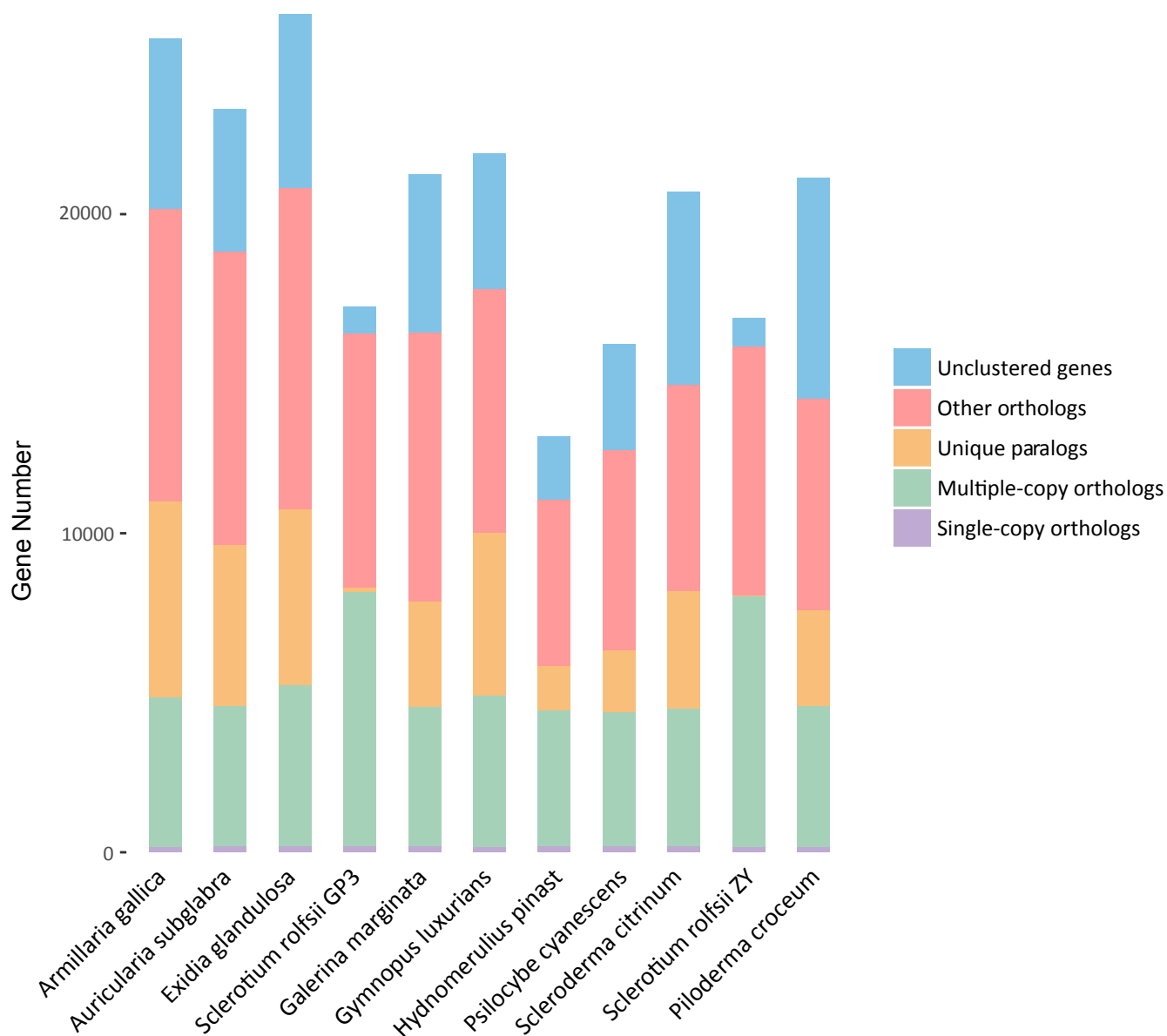

Supplement: Supplementary file 6 — Additional file 6: Figure S6. Analysis of orthologs of two S. rolfsii strains and other species in Agaricomycetes [file 12864_2021_7534_MOESM6_ESM.pdf]
