# Supplementary material for: Genome sequencing and comparative genomic analysis of highly and weakly aggressive strains of Sclerotium rolfsii, the causal agent of peanut stem rot
Source: BMC Genomics. 2021 Apr 16;22:276. doi: 10.1186/s12864-021-07534-0 (PMC8052761; doi:10.1186/s12864-021-07534-0)
Supplement: Supplementary file 3 — Additional file 3: Figure S3. KOG distribution of predicted proteins of S. roflsii GP3 and ZY [file 12864_2021_7534_MOESM3_ESM.pdf]

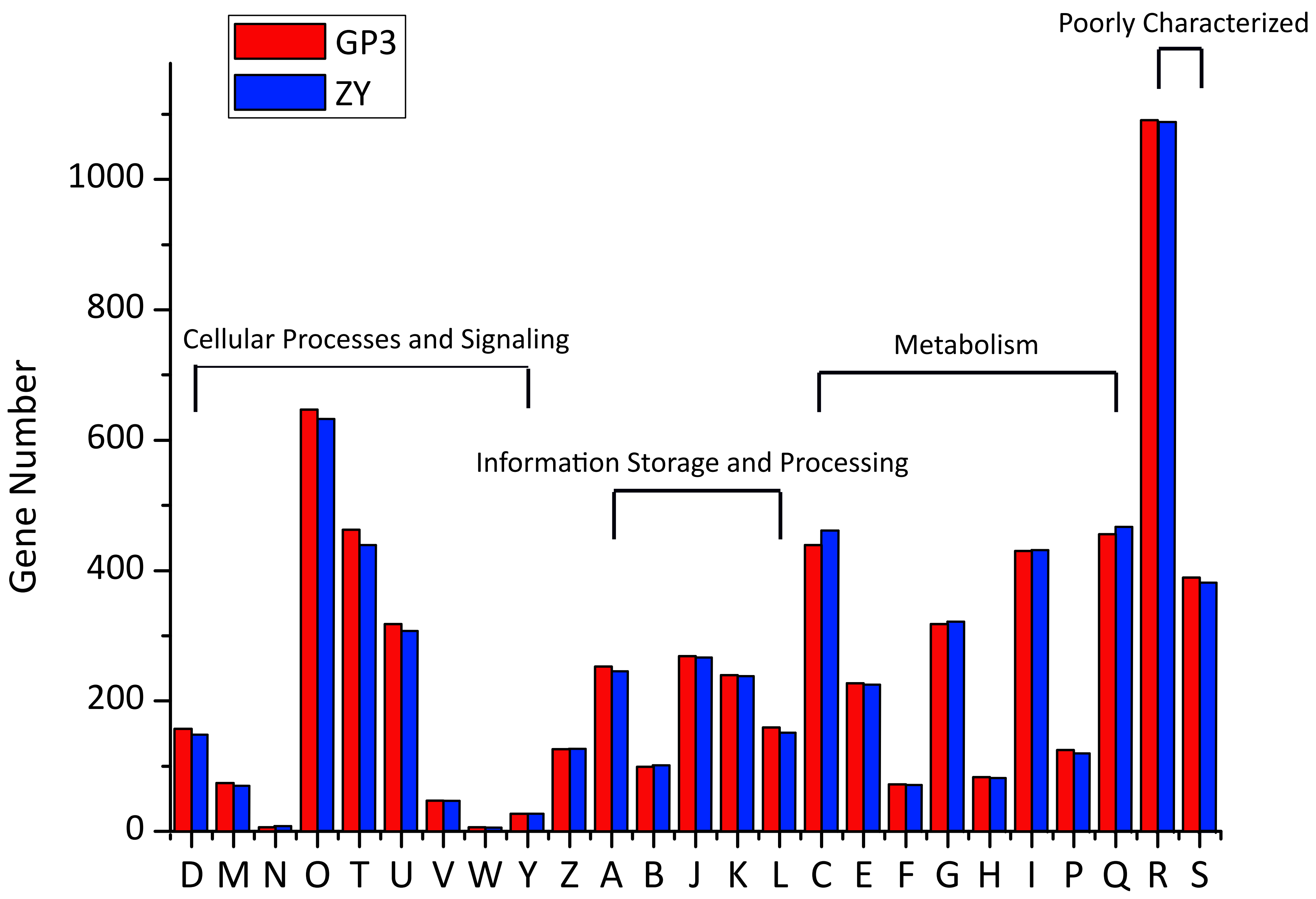

[A] RNA processing and modification
[B] Chromatin structure and dynamics
[C] Energy production and conversion
[D] Cell cycle control, cell division, chromosome partitioning
[E] Amino acid transport and metabolism
[F] Nucleotide transport and metabolism
[G] Carbohydrate transport and metabolism
[H] Coenzyme transport and metabolism
[I] Lipid transport and metabolism
[J] Translation, ribosomal structure and biogenesis
[K] Transcription
[L] Replication, recombination and repair
[M] Cell wall/membrane/envelope biogenesis
[N] Cell motility
[O] Post-translational modification, protein turnover, and chaperones
[P] Inorganic ion transport and metabolism
[Q] Secondary metabolites biosynthesis, transport, and catabolism
[R] General function prediction only
[S] Function unknown
[T] Signal transduction mechanisms
[U] Intracellular trafficking, secretion, and vesicular transport
[V] Defense mechanisms
[W] Extracellular structures
[Y] Nuclear structure
[Z] Cytoskeleton
