# Supplementary material for: Genome sequencing and comparative genomic analysis of highly and weakly aggressive strains of Sclerotium rolfsii, the causal agent of peanut stem rot
Source: BMC Genomics. 2021 Apr 16;22:276. doi: 10.1186/s12864-021-07534-0 (PMC8052761; doi:10.1186/s12864-021-07534-0)
Supplement: Supplementary file 7 — Additional file 7: Figure S7. The pipeline for putative secretomes and effectors analysis of S. roflsii GP3 and ZY [file 12864_2021_7534_MOESM7_ESM.pdf]

Putative proteins
(GP3, 17097; ZY, 16743)

↓

Singnal P 4.0
(GP3, 1522; ZY, 1400)

↓

Protcomp 9.0
(GP, 639; ZY, 781)

↓

TMHMM
(GP3, 631; ZY, 637)

↓

Big -PI
(GP3, 600; ZY, 602)

↓

TargetP
(Seretome: GP3, 536; ZY, 551)

↓

Effector P
(GP3, 50; ZY, 46)

↓

Protein size 50~300, Cys ≥4

(Putative effectors: GP3, 30; ZY, 27)
